# Complete Genome Sequence of a Novel *Lactobacillus paracasei* TK1501 and Its Application in the Biosynthesis of Isoflavone Aglycones

**DOI:** 10.3390/foods11182807

**Published:** 2022-09-12

**Authors:** Yufeng Xie, Yingxue Wang, Yang Han, Jing Zhang, Shumei Wang, Shuwen Lu, Haikuan Wang, Fuping Lu, Longgang Jia

**Affiliations:** 1 College of Food Science and Engineering, Harbin University, Harbin 150086, China; 2 Food Processing Institute, Heilongjiang Academy of Agricultural Sciences, Harbin 150086, China; 3 College of Food Science and Engineering, Tianjin University of Science and Technology, Tianjin 300457, China; 4 College of Pharmacy, Qilu Medical University, Zibo City 255300, China

**Keywords:** complete genome sequence, *Lactobacillus paracasei* TK1501, isoflavone aglycones, β-glucosidase

## Abstract

*Lactobacillus* strains are considered safe and healthy probiotics for manufacturing “natural food” products; this is due to their ability to produce bioactive compounds that reduce the incidence of various human diseases. *Lactobacillus paracasei* TK1501 is a novel probiotic strain isolated from naturally fermented congee; and can produce a high yield of genistein, one of the most widely studied isoflavone aglycones with plenty of physiological functions. To better understand the molecular basis of isoflavone aglycones biosynthesis, the complete 2,942,538 bp genome of *L. paracasei* TK1501 was sequenced and assembled; a group of genes that are involved in isoflavone aglycones production were identified. Of note, a β-glucosidase was analyzed in the *L. paracasei* TK1501. Moreover, we also found that *L. paracasei* TK1501 could be used in soymilk fermentation; which would remarkably increase the contents of genistein, daidzein, and glycitein. This work was meaningful to the application of *L. paracasei* TK1501 and the molecular mechanism analysis of isoflavone aglycones biosynthesis in *Lactobacillus* strains.

## 1. Introduction

Soy isoflavone aglycones are a class of phytoestrogens with highly bioactive properties on lots of cellular processes and human diseases [1,2], including osteoporosis, cardiovascular disease [3], type 2 diabetes [4,5], and cancers [6]. Compared to the related glycosides, the isoflavone aglycones are with higher bioactivity due to their lower molecular weight and unimpeded gut absorption [7]. Therefore, there is a growing interest in the production of “natural food” products containing high levels of isoflavone aglycones. Glycosylated isoflavones can be converted to isoflavone aglycones according to a hydrolysis reaction using β-glycosidases. In addition, β-glycosidases commonly exist in plants and microorganisms, especially in lactic acid bacteria. Lactic acid bacteria are normally found in the gastrointestinal tract, and are frequently used as probiotics in fermented dairy products such as soybean milk [8]. *Lactobacillus paracasei* (*L. paracasei*) is a member of the normal gut microbiota and has the ability to produce genistein. Therefore, it is one of the most promising candidates for the manufacture of safe and healthy natural food such as fermented soymilk [9,10,11]. *L. paracasei* strains have already been used for in situ fortification of certain foods in the dairy industry. Thus, it would be beneficial to identify novel *L. paracasei* strains with higher yields of isoflavone aglycones. Herein, we have isolated a novel *L. paracasei* TK1502 from naturally fermented congee. Moreover, *L. paracasei* TK1502 could produce a high yield of genistein, an important isoflavone aglycone with many functions. In order to gain a better insight into the molecular mechanism of the isoflavone aglycones biosynthesis, the complete genome of *L. paracasei* TK1502 was sequenced; and a comparative *β-glucosidase* gene analysis was performed between *L. paracasei* TK1502 and several other bacterial strains. We also probed the beneficial effects of *L. paracasei* TK1502 in the application of fermented soymilk.

## 2. Materials and Methods

### 2.1. Strains and Data Storage

*L. paracasei* TK1501 was stored in the China General Microbiological Culture Collection (CGMCC) with an accession number of CGMCC13130. The complete genome sequences of *L. paracasei* TK1501 were deposited in the NCBI database under the accession number of CP017716.

### 2.2. DNA Extraction

The *lactobacillus* strains in this study were isolated from traditional fermented congee obtained from Inner Mongolia, China. These bacteria were cultured at 37 °C in brain-heart infusion broth under anaerobic conditions (80% N_2_, 10% H_2_, and 10% CO_2_) for 24 h. *Lactobacillus* selected for identification by a sequence analysis of 16S rDNA were collected by centrifugation (12,000× *g*, 5 min) from cultures that were incubated for 24 h at 37 °C; and their DNA was extracted using a previously described method, with some modifications [12].

### 2.3. PCR Amplification and Sequencing

The polymerase chain reaction was used to amplify the 16S rDNA gene using the primers (16S-S Forward:5′-AGAGTTTGATCCTGGCTCAG-3′; 16S-R Reverse:5′-TACGGCTACCTTGTTACGACTT-3′). The polymerase chain reaction conditions were as follows: 95 °C for 5 min, followed by 30 cycles of 1 min at 95 °C, 1 min at 55 °C, and 1 min at 72 °C, followed by 5 min at 72 °C. The complete genome was sequenced using a whole genome shotgun strategy combining next-generation sequencing and third-generation sequencing based on Illumina MiSeq paired-end and PacBio standard sequencing technology.

### 2.4. Phylogenetic Analysis and Gene Annotation

The reads were de novo assembled using Newbler and HGAP 2.3.0; and integration of the assembled contigs was carried out using Mummer and Pilon. Automated gene prediction and annotation of the whole genome sequences were carried out using Glimmer v.3.0 and the NCBI Prokaryotic Genome Annotation Pipeline, respectively. Ribosomal RNA genes were identified using RNAmmer v.1.2, and tRNA genes were predicted using a tRNA scan-SE v.1.3.1 [13]. Each gene was functionally assigned to a category using the following databases: Evolutionary Genealogy of Genes: Non-supervised Orthologous Groups (eggNOG) [14]; Kyoto Encyclopedia of Genes and Genomes (KEGG) [15]; Swiss-Prot, Gene Ontology (GO) [16]; and the NCBI Non-Redundant Protein Database (NR) [17]. Cycle maps of the genomes were made with cgview and compiled by Photoshop CS [18]. The phylogenetic tree was constructed using MEGA 6.0. HMMER 3.0 software [19], which was used to predict the carbohydrate-active enzyme (CAZy) genes from the genome sequence. The critical E-value was set as 1 × 10^6^ when the sequence was ≥ 80 amino acids; and was set as 1 × 10^3^ when the sequence was <80 amino acids.

### 2.5. β-Glucosidase Activity Analysis of L. paracasei TK1502

We analyzed the β-glucosidase catalytic capacity of *L. paracasei* TK1502 after fermentation for 24 h, following our previously reported method [20]. One unit (U) of β-glucosidase activity was defined as the amount of enzyme that generated 1 μM p-nitrophenol (pNP) from the substrate of p-nitrophenyl-β-D-glucopyranoside (pNPG) per min. After being fermented at 37 °C for 24 h, the supernatant of *L. paracasei* TK1502 was removed at 15,000× *g* for 10 min. The cell pellet was suspended in 10 mM PBS buffer (pH 7.4) and sonicated for 20 min (on 1 s, off 1 s); the supernatant was obtained after being centrifuged at 4 °C, 15,000× *g* for 10 min. Then, 5 mM pNPG was mixed with a 5 mL *L. paracasei* TK1502 cell lysis solution and incubated at 37 °C for 30 min. The reaction was stopped by adding 250 μM 0.2 M Na_2_CO_3_. Then, the absorbance of samples at 410 nm was tested using a UV–vis spectrophotometer.

### 2.6. Application of L. paracasei TK1502 in the Fermentation of Soymilk

*L. paracasei* TK1502 or other bacterial strains were cultured at 37 °C in 10 mL of sterile soymilk for 24 h to prepare the seeds. After calculating the concentration of the bacteria, 1 × 10^7^ CFU/mL of strains seed was transferred into an 80 mL fresh sterile soymilk medium and cultured at 37 °C for 24 h. The sterile soymilk medium without any bacterial strains was defined as the blank control; it was also treated in the same incubation conditions as the experimental group. The fermented solution was lyophilized, and stored at −80 °C until use. In total, 0.5 g of the lyophilized samples were dissolved in a small amount of DMSO, and re-solved in 5 mL of 80% methanol. The sample solutions were incubated by shaking at 60 °C for 2 h, and then centrifuged at 12,000 rpm, 4 °C for 20 min. The supernatant of each sample was filtered using a 0.22 μm membrane for measurement.

High-performance liquid chromatography (HPLC) was used to test the changes of the soy isoflavones and isoflavone glycosides. Herein, we mainly measured three soy isoflavones, including genistin, daidzin, and glycitin; and their hydrolysis products of genistein, daidzein, and glycitein, respectively. All of the standard substances were purchased from Sigma-Aldrich (St. Louis, MO, USA). A NovaPak C18 column (250 × 4.6 mm, 5 μm, Waters, Milford, MA, USA) was used for the separation, and a photodiode array detector (PDA-100, Dionex) was used for the detection process. Mobile phase A was water with 0.1% acetic acid, and phase B was methanol with 0.1% acetic acid. The flow rate was 1 mL/min and the other parameters were as follows: 0 min, 20% phase B; 8 min, 60% phase B; 18 min, 95% phase B; 19 min, 20% phase B; 24 min, 20% phase B; the column temperature was 27 °C; and the wavelength was 260 nm.

## 3. Results and Discussion

### 3.1. Genome Organization and Base Composition

**Figure 1:**
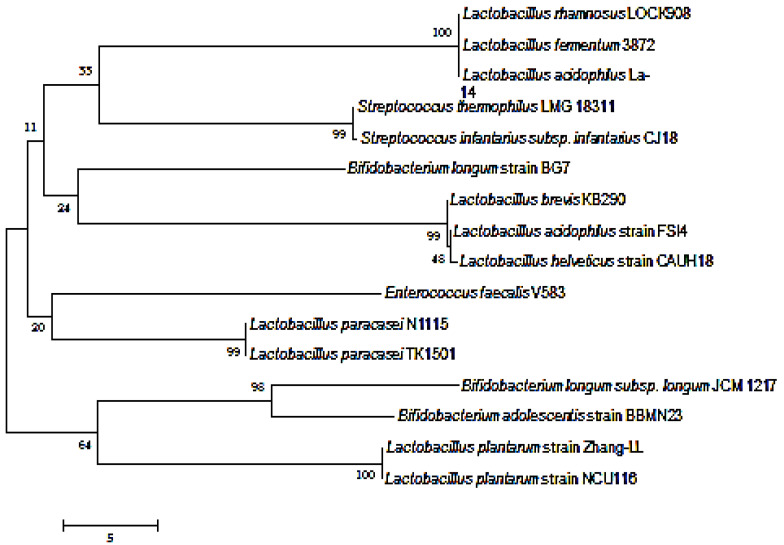
Phylogenetic tree of *L. paracasei* TK1501. The phylogenetic tree was constructed using MEGA 6.0.

In this present study, *L. paracasei* TK1501 was isolated from naturally fermented congee. This strain is a facultative anaerobic, rod-shaped and Gram-positive bacteria; the colonies of which are milky white, round, opaque, and the surface of which is smooth and with a convex elevation. D-glucose, fructose, lactose, maltose, D-mannose, and cellose can be used as carbon source; in addition, peptone, beef extract, yeast extract, ammonium nitrate, ammonium sulfate, and ammonia chloride can be used as nitrogen source for the *L. paracasei* TK1501. Furthermore, *L. paracasei* TK1501 is catalase-negative and protease-positive; also, it cannot digest gelatin or produce sulfuretted hydrogen. *L. paracasei* TK1501 showed a ≥99% nucleotide sequence similarity to other *Lactobacillus* strains deposited in the GenBank database based on 16S rRNA gene analysis. Phylogenetic analysis showed that TK1501 formed a distinct branch with *L. paracasei* strains (Figure 1), and was most closely related to *L. paracasei* N1115. TK1501; it was therefore preliminarily designated as a *L. paracasei* strain. Furthermore, the capacity of *L. paracasei* TK1501 on the production of genistein by fermenting soymilk was compared with several other *lactobacillus* strains. After fermentation using *L. paracasei* TK1501, the contents of genistein in the soymilk was increased 25.34-fold compared with the blank control group that was fermented without any bacterial strains. Meanwhile, *L. paracasei* TK1501 had a much higher increase in the amount of times with respect to the genistein in the fermented soymilk than in any of the other bacterial strain groups (Table 1).

**Figure 2:**
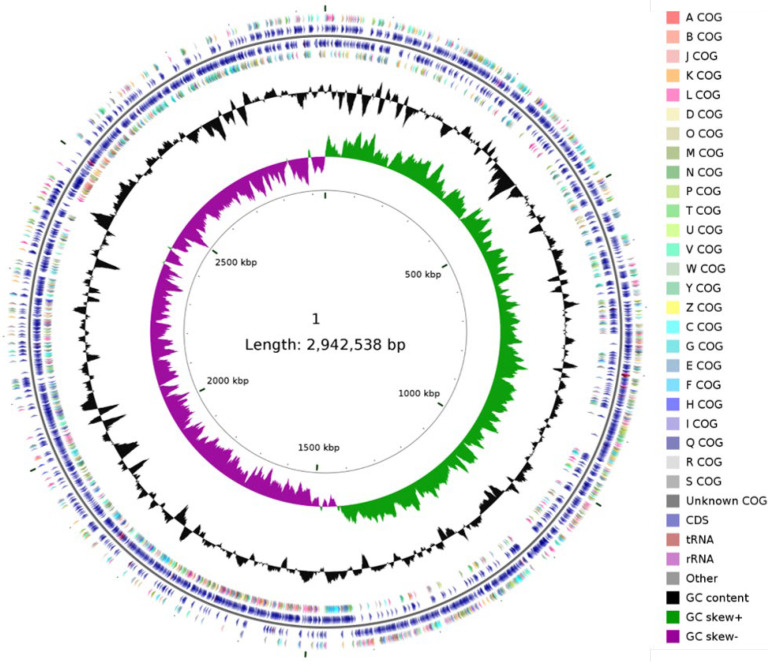
Circular genome map of *L. paracasei* TK1501.

Complete genome sequencing and genomic analysis of bacterial strains can provide functional information. To gain insights into the genetic elements involved in the production of genistein, we sequenced the entire genome of *L. paracasei* TK1501. A total of 693,261 reads were generated, with an average length of 6768 nucleotides. As shown in Figure 2 and Table 2, the complete genome of TK1501 is composed of a single circular chromosome, 2,942,538 bp in length. There were 2947 protein-coding genes, 59 tRNA genes, and 15 rRNA genes, along with 40 other non-coding RNA regions. The predicted G + C content was 46.54%, and no plasmids were identified.

### 3.2. Analysis of Carbohydrate-Active Enzymes

The carbohydrate-active enzymes (CAZy) database mainly contains glycoside hydrolases (GHs), glycosyl transferases (GTs), polysaccharide lyases (PLs), carbohydrate esterases (CEs), and auxiliary activities (AAs) [21]. Moreover, carbohydrate-binding modules (CBMs) are also included. Genes of CAZys in the genome sequences were predicted with HMMER (version 3.0). The analysis results were shown in Table 3; the numbers of CAZy genes are 77, which is 2.61 percent of the whole genome. In addition, GHs and GTs were the main enzymes of the predicted CAZy genes.

### 3.3. Comparative Analysis of β-Glucosidase from L. paracasei TK1501

β-glucosidases are the key enzyme during genistein biosynthesis, and belong to GHs; in addition, they have been reported to have the function to hydrolyse isoflavones [22]. Firstly, we analyzed the β-glucosidase catalytic capacity of *L. paracasei* TK1502 using pNPG as the substrate. The results showed that the β-glucosidase activity of *L. paracasei* TK1502 was 17.45 U/mL; this was remarkably higher than many probiotic stains or other microorganisms, such as *L. perolens* FI10842 (49.10 mU/mL) [23], *L. bulgaricus CFR2028* (~160 mU/mL) [24], *A. niger* NRRL 3112 (9.30 U/mL) [25], *T. atroviride* TUB F-172 (11.70 U/mL) [26], and so on. Therefore, *L. paracasei* TK1502 was a good source of β-glucosidases that possessed an excellent β-glucosidase catalytic capacity.

**Figure 3:**
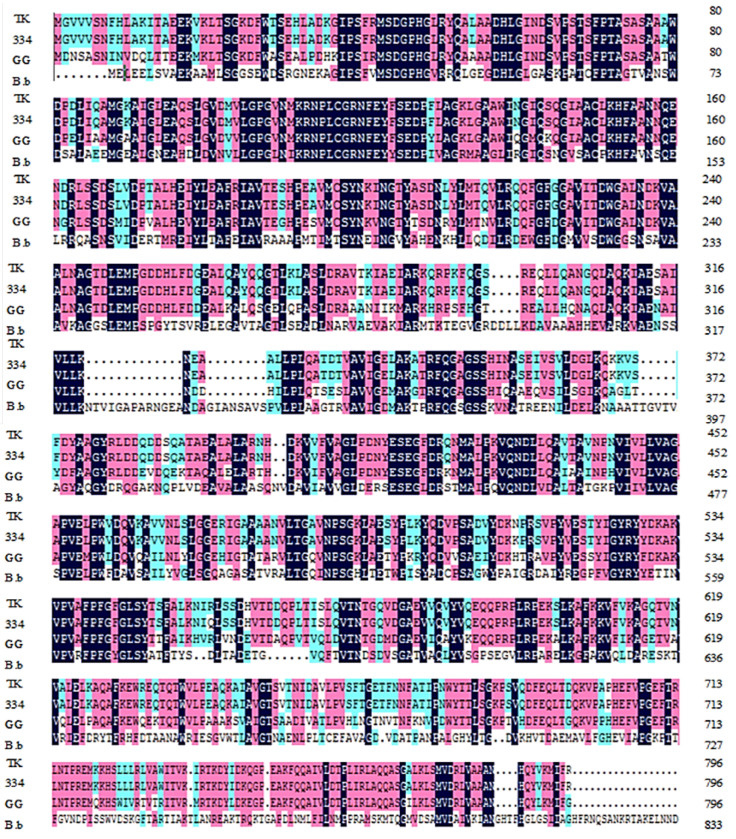
Comparison of β-glucosidase amino acid sequences from *L. paracasei* TK1501 (TK) with *L. casei* ATCC334 (334), *L. rhamnosus* GG (GG), and *Bifidobacterium* B.b (B.b). The navy-blue means consistent residues in all four enzymes, purple means consistent residues in three enzymes, and light blue means consistent residues in two enzymes.

In order to analyze the genomic properties of β-glucosidase in *L. paracasei* TK1501, the β-glucosidase amino acid sequence from *L. paracasei* TK1501 was compared with the corresponding sequences from several other available bacterial strains of *Lactobacillus casei* ATCC334, *Lactobacillus rhamnosus* GG, and *Bifidobacterium* B.b. The TK1501 sequence showed a 99.8% similarity to that of *L. casei* ATCC334; however, it had only a 76.4% and 36.6% similarity to the sequences from *L. rhamnosus* GG and *Bifidobacterium* B.b, respectively (Figure 3). Together, our findings suggest that the production of genistein is potentially related to several important genetic differences between TK1501 and closely related strains.

### 3.4. The Isoflavone Aglycones in Soymilk Were Increased after Fermentation Using L. paracasei TK1502

**Figure 4:**
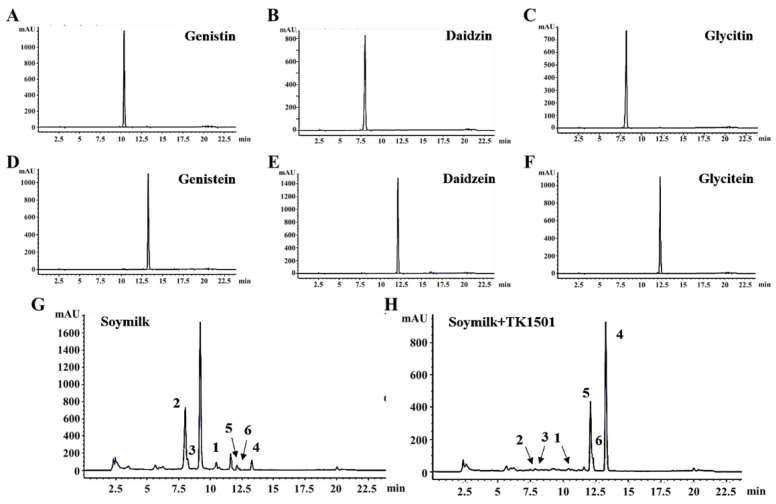
Analysis of isoflavones and related isoflavone aglycones changes in the fermentation of soymilk by *L. paracasei* TK1501 using HPLC. Standard molecules of isoflavones, including (**A**) genistin, (**B**) daidzin, and (**C**) glycitin; and their corresponding aglycones of (**D**) genistein, (**E**) daidzein, and (**F**) glycitein. Fermented soymilk (**G**) without and (**H**) with *L. paracasei* TK1501; in which compounds 1–6 were genistin, daidzin, and glycitin; and genistein, daidzein, and glycitein, respectively.

Since β-glucosidases have a broad catalytic capacity for the hydrolysis of many isoflavones to produce isoflavone aglycones, we further measured the changes of three important isoflavones, including genistin, daidzin, and glycitin; and their corresponding aglycones of genistein, daidzein, and glycitein after fermentation using *L. paracasei* TK1501 by HPLC. The HPLC data of the standard molecules of genistin, daidzin, and glycitin; and their aglycones of genistein, daidzein, and glycitein are shown in Figure 4A–F; herein, all the retention times of the isoflavones were less than their aglycones. The degradation rates of genistin, daidzin, and glycitin in the soymilk after fermentation using *L. paracasei* TK1501 were (94.87 ± 0.06), (83.57 ± 0.14), and (97.05 ± 0.08)%, respectively (Table 4). Compared to the soymilk without *L. paracasei* TK1501, the contents of genistein, daidzein, and glycitein were remarkably increased from (8.04 ± 0.06), (2.24 ± 0.09), and (2.43 ± 0.08) μg/g to (211.65 ± 1.27), (25.39 ± 0.13), and (109.10 ± 1.19) μg/g, respectively. The results proved that the *L. paracasei* TK1501 could be applied in the fermentation of soymilk, and could increase the contents of several important isoflavone aglycones; this could be easily absorbed and beneficial to ameliorate many human conditions, including diabetes, obesity, cancers, hypertension, and so forth [2,7,27].

## 4. Conclusions

In this study, we reported the complete sequence of a novel probiotic strain isolated from the naturally fermented congee, *L. paracasei* TK1501. The complete genome of *L. paracasei* TK1501 was 2,942,538 bp. A group of isoflavone aglycone production-related genes, including the β-glucosidase gene, were analyzed and identified in the *L. paracasei* TK1501. Moreover, the contents of isoflavone aglycones, including genistein, daidzein, and glycitein in the soymilk, were remarkably increased after fermentation using *L. paracasei* TK1501. The complete genome sequence of *L. paracasei* TK1501 will enrich its application, and help us to better understand the molecular basis of isoflavone aglycones biosynthesis in the *lactobacillus*.

## Figures and Tables

**Table 1:** Comparison of genistein production in the soymilk after fermentation using the different *lactobacillus* strains.

**Strains**	**Increased Folds**
*Lactobacillus paracasei* TK1501	25.34
*Lactobacillus rhamnosus* CRL981	4.66
*Lactobacillus rhamnosus* D7	17.06
*Lactobacillus paracasei* BCRC 14023	1.10
*Lactobacillus delbrueckii* 01181	8.36
*Lactobacillus plantarum* 00144	7.15
*Streptococcus thermophilus* CCRC14085	2.59
*Bifidobacterium breve* K-101	3.01
*Lactobacillus acidophilus* BCRC 10695	1.06

**Table 2:** Features of the *L. paracasei* TK1501 genome.

**Attributes**	**Values**
Genome size (bp)	2,942,538
GC content (%)	46.54%
Plasmid	0
rRNAs	15
tRNAs	59
Other ncRNAs	40
Proteins	2947

**Table 3:** Analysis of CAZy in *L. paracasei* TK1501.

**Property**	**Numbers of Genes**	**Percentage (%)**
GHs	36	1.22
GTs	26	0.88
PLs	0	0.00
CEs	9	0.31
AAs	4	0.14
CBMs	2	0.07
Total	77	2.61

**Table 4:** The degradation rate of isoflavones in the *L. paracasei* TK1501 fermented soymilk.

**Isoflavones**	**Degradation Rate (%)**
Genistin	94.87 ± 0.06
Daidzin	83.57 ± 0.14
Glycitin	97.05 ± 0.08

## Data Availability

The data presented in this study are available on request from the corresponding author.
